# Irreversible electroporation in patients with liver tumours: treated-area patterns with contrast-enhanced ultrasound

**DOI:** 10.1186/s12957-020-02083-4

**Published:** 2020-11-23

**Authors:** Linyu Zhou, Shanyu Yin, Weilu Chai, Qiyu Zhao, Guo Tian, Danxia Xu, Tian’an Jiang

**Affiliations:** 1 Department of Ultrasonography, The First Affiliated Hospital, College of Medicine, Zhejiang University, No. 79 Qingchun Road, Hangzhou, 310003 Zhejiang Province P.R. China; 2 Department of Hepatobiliary and Pancreatic Surgery, The First Affiliated Hospital, College of Medicine, Zhejiang University, No. 79 Qingchun Road, Hangzhou, 310003 Zhejiang Province P.R. China; 3 Collaborative Innovation Center for Diagnosis and Treatment of Infection Diseases, The First Affiliated Hospital, College of Medicine, Zhejiang University, Hangzhou, P.R. China

**Keywords:** Irreversible electroporation, Liver tumours, Contrast-enhanced ultrasound, Ablation, Ultrasonography

## Abstract

**Background:**

Familiarity with post-IRE imaging interpretation is of considerable importance in determining ablation success and detecting recurrence. CEUS can be used to assess the tumour response and characteristics of the ablation zone. It is of clinical interest to describe the ultrasonographic findings of liver tumours after irreversible electroporation (IRE) percutaneous ablation.

**Methods:**

A prospective study of 24 cases of malignant liver tumours (22 cases of primary liver tumours and 2 cases of liver metastases) treated by IRE ablation was conducted. Two inspectors evaluated the ablation zone in a consensus reading performed immediately, 1 day, and 1 month after IRE ablation. The gold standard method, magnetic resonance imaging (MRI), was used to evaluate the effectiveness of the treatment at 1 month.

**Results:**

Immediately after IRE ablation and up to 1 month later, the ablation zones gradually changed from hypo-echogenicity to hyper-echogenicity on conventional ultrasound and showed non-enhancement on contrast-enhanced ultrasound (CEUS). One month after IRE ablation, CEUS and MRI results were highly consistent (κ = 0.78, *p* < 0.05).

**Conclusions:**

We conclude that CEUS may be an effective tool for assessing post-IRE ablation changes after 1 month. CEUS enables the depiction of tumour vascularity in real time and serves as an easy, repeatable method.

## Introduction

Liver cancer is the fourth leading cause of cancer-related deaths in the world [1]. Most patients are not eligible for radical surgical resection at the time of diagnosis. Radiofrequency ablation (RFA), microwave ablation (MWA) or cryoablation is potentially curative in select patients. However, the efficacy of ablation is limited by the size, number and location of the lesion. As a result, the effectiveness and safety of these techniques are limited for lesions adjacent to important structures, such as the bile duct, portal vein, and gastrointestinal tract. For patients with such lesions, irreversible electroporation (IRE) is considered an alternative treatment. Compared to other local ablation techniques, IRE is a promising technique. IRE is a non-thermal ablation method that induces tumour necrosis by inducing apoptosis and cell death. IRE treatment generates electric pulses that alter the cell membrane’s electrical potential, leading to small nanopores and contributing to apoptosis [2]. The efficacy of IRE is unaffected by the so-called heat-sink effect. Thus, IRE is currently being applied experimentally and clinically in a wide range of tissues [3, 4].

Contrast-enhanced ultrasound (CEUS) allows continuous real-time observation of lesion tissue enhancement in arteries, portal veins, and advanced stages. Thus, CEUS can dynamically assess blood flow and tissue perfusion. The feasibility of using CEUS to assess ablation areas has been reported in some preliminary animal and clinical studies [5, 6].

CEUS can be used to assess the tumour response and the characteristics of the ablation zone. Only limited data are available on the CEUS imaging characteristics of the ablation zones after IRE ablation in humans. Previous studies on lesions after IRE have focused on the imaging manifestations of CT and MRI [7–9], though there are few studies on the performance of lesions after IRE in CEUS. For example, Lin et al. and Rennert et al. used animal experiments to explore the changes in lesions within 2 h and 24 h after IRE, respectively [10, 11] and the clinical case study of Niessen et al. focused on changes of lesions from 6 weeks to 1 year after IRE [12]. To understand the dynamic changes of lesions on CEUS from a few hours to 1 month after IRE, we designed and conducted this study to provide a basis for postoperative imaging follow-up. After IRE ablation, cell death occurs with electroporation of the cell membrane and changes in histomorphology. Thus, the assessment is different from those for other ablation methods. Familiarity with post-IRE imaging interpretation is of considerable importance in determining ablation success and detecting recurrence.

The present study focuses on specific imaging characteristics of hepatic tumours obtained by CEUS with an intravenous contrast agent immediately, 1 day, and 1 month after US-guided percutaneous IRE ablation. Magnetic resonance imaging (MRI) was performed 1 month after ablation to investigate the therapeutic efficacy.

## Materials and methods

This study was conducted with the approval of our institutional review board. The research conformed to the Declaration of Helsinki. Before receiving CEUS treatment, patients signed an informed consent form for imaging data analysis.

### Patients

We conducted a single-centre prospective study to evaluate and describe the CEUS imaging findings of liver tumours after percutaneous IRE ablation. From May 2016 to June 2019, 21 patients were treated with IRE at our institution. All patients met the following inclusion criteria: unresectable tumours; tumours unsuitable for thermal ablation because of close proximity to major veins; and biliary and venous systems of the liver that would cause heat-sink effects or collateral damage; these definitions were similar to the difficult location definitions set by Wei Yang et al. [13]. A history of hemi-hepatectomy was not considered a contraindication in the present study. Thermal ablation or catheter chemical ablation was not a contraindication. The exclusion criteria were a history of epilepsy or arrhythmia and the presence of an implanted cardiac pacemaker or metal biliary stent.

### IRE procedures

All steps were performed under general anaesthesia using the NanoKnife IRE system (Angiodynamics Inc., Latham, NY, USA). A doctor with more than 5 years of experience in US-guided interventional procedures and IRE performed all IRE procedures. The IRE generator was programmed following the manufacturer’s instructions. Based on the pre-IRE image, the NanoKnife System calculated related parameters, namely, the number of electrodes, the expected ablation area, the number of electrodes, and the distance between the electrodes. The ablation procedure is the same as that described in the previous study. The required number of needles was selected according to the nodule size. The applied voltage was 1800–3000 V (pulse length 70–90; pulse repetition number 90–270). Ablation was performed under ultrasound guidance.

### Imaging procedure

US scanning was performed to assess the locations, sizes and margins of the tumours before ablation. The first image acquisition was conducted after the intervention (immediately after ablation), and follow-up imaging was performed to assess the development of the ablation zone.

CEUS was used to evaluate the effect of IRE. All CEUS studies were performed using Mylab 90 (Esaote, Italy) or ultrasound scanners equipped with 3.5–6-MHz convex transducers. The US contrast agent used was SonoVue (Bracco SpA, Milan, Italy). After injecting 2.4 ml of SonoVue agent, 5 mL of 0.9% saline was injected. Immediately after injection the ultrasound contrast agent, a dual-B mode image was acquired. Simultaneously, a timer was started. According to previous studies, we defined three phases: the arterial phase (15–30 s), the portal venous phase (31–120 s), and the late phase (121 s and later). The ablation zone was observed continuously for 5 min. The entire image for inspection was recorded digitally and stored on the hard disk of the US scanner for subsequent analysis.

All CEUS studies were performed before ablation and immediately, 1 day, and 1 month after tumour ablation to evaluate the characteristics of the IRE ablation zone. MRI was conducted 1 month after ablation to investigate the therapeutic efficacy.

### CEUS analysis

One operator with 10 years of experience with CEUS performed all the CEUS examinations during the course of the study. Data were analysed and defined by consensus between two doctors. If different opinions arose, the reviewers jointly re-assessed the saved images and then reached a consensus. The presence or absence of tumour enhancement on the immediate CEUS image was recorded. The following imaging features were assessed: echogenicity, the boundary and the enhancement pattern of the IRE ablation zones.

### Statistical methods

A p value of 0.05 was considered statistically significant. Descriptive statistics were used to present the results as absolute numbers (n), the means and standard deviations (SDs), or percentages. We compared the efficacy of CEUS assessment with MRI by Cohen's κ values [14]. In addition, sensitivity, specificity, and positive and negative predictive values were all calculated. All statistical analyses were performed using SPSS software (version 23.0).

## Results

The patient group included 21 patients (24 ablation lesions) aged 31–86 years (mean 59.6 ± 12.7 years). The identity of the tumour could be histologically confirmed in 15 cases, while 9 lesions were diagnosed based on imaging. The histologic findings showed hepatocellular carcinoma (HCC) in 13 lesions and metastases of gastrointestinal tumours in 2 lesions.

### Immediate post-procedural assessment

**Fig. 1:**
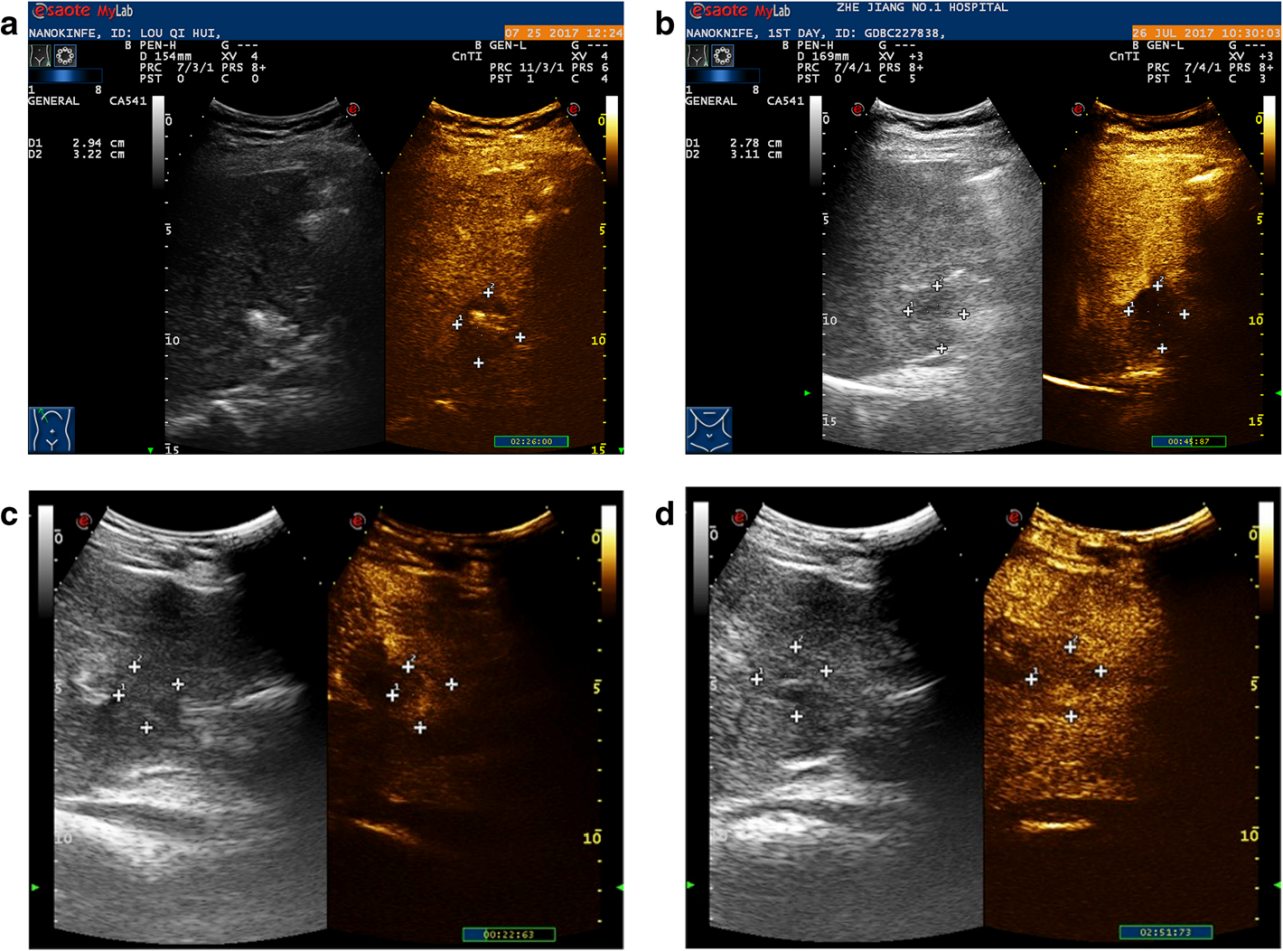
**a** Immediate post-IRE CEUS demonstrated complete non-enhancement in the ablation zone. The boundary of the non-enhanced area was clearly outlined. **b** A follow-up scan one day after IRE also showed complete non-enhancement. **c** A 46-year-old man presented with hepatic carcinoma. One month after IRE ablation, a hyper-enhanced lesion was observed in the early arterial phase. **d** The hyper-enhanced foci washed out during the late arterial phase

On conventional US performed immediately after IRE treatment, the ablated zones were either hypo-echoic (17/24, 71%) or iso-echoic (7/24, 29%). The boundaries were unclear, and the echogenicity of the boundary was heterogeneous. On CEUS, in 4 of the 24 ablated areas (17%), no enhancement was observed, and the boundary was clearly outlined (Fig. 1a). Enhancement of the IRE ablation zone was observed in 20 of the 24 cases (83%): 1 ablation zone showed slight hyper-enhancement in the early arterial phase that was heterogeneous and washed out in the late arterial phase, and 19 ablation zones showed hypo-enhancement in the arterial phase. The boundaries between the enhanced and non-enhanced zones were clear.

### One-day follow-up

B-mode imaging of the ablated zones performed 1 day after IRE treatment showed hyper-echoic foci (13/24, 54%), iso-echoic foci (5/24, 20%), hypo-echoic foci (6/24, 25%). The boundaries were clear. On CEUS, 16 of 24 ablated areas showed no enhancement at all (67%) (Fig. 1b). Enhancement of the ablation zone was observed in 8 of the 24 zones (33%). One ablation zone showed slight hyper-enhancement in the early arterial phase, which washed out in the late arterial phase, while others showed hypo-enhancement in the arterial phase. During the portal venous and late phases, the enhanced foci showed hypo-enhancement.

### One-month and later follow-up examinations

On conventional US performed 1 month after IRE treatment, 22 ablated zones showed hyper-echoic foci with clear boundaries (22/24, 92%), and 2 ablated zones were still hypo-echoic (2/21, 10%). Complete non-enhancement was documented in 19 of the 24 ablation zones (79%). One ablation zone showed slight hyper-enhancement in the early arterial phase that washed out in the late arterial phase (Fig. 1c and d). Another 4 zones showed quick wash-in and quick wash-out patterns on CEUS. No evidence of peripheral contrast enhancement could be found during the arterial phase or during the portal venous phase.

One month after IRE ablation, no evidence of recurrence was found by MRI in 17 ablation zones (17/24, 71%), while recurrence was identified in 7 zones (7/24, 29%). Substantial agreement (κ = 0.78; *p* < 0.05) was observed between the results obtained with CEUS and those obtained with MRI. With MRI as the gold standard, the sensitivity, specificity, positive predictive value and negative predictive value of CEUS were 71.4%, 100%, 100%, and 89.5%, respectively.

## Discussion

IRE has attracted increasing interest because the non-thermal properties of this method [15] permit ablation of tumours adjacent to vital structures [16]. Previous studies have shown that the ultrasound findings of IRE applied to the liver will change from a few seconds to at least a few hours with time [3, 17, 18]. Our results show the evolution of post-IRE ablation patterns over a clinically relevant period.

As noted in an animal-based experiment, a strong association exists between conventional US and histopathology [5]. In our study, on conventional US, the IRE ablation zones of most patients appeared as a developing hypo-echoic area that demonstrated an increasingly hyperechogenic ablation zone starting one day after the procedure.

Our US findings after IRE ablation evolved sequentially over time. The findings showed good correlations with those in previous studies [5, 19]. In the study, specimens obtained immediately after ablation showed that the enlarged oedematous sinusoidal space was mainly filled with fluid, and almost no haemorrhagic infiltration was observed. As time passed, the haemorrhagic infiltrate became more dominant, which might be due to the widened fluid-filled sinusoids at the beginning of the procedure. The authors attribute this hyper-echogenicity to red blood cell accumulation over time. The degree of erythrocyte infiltration was qualitatively related to echogenicity. Lee et al. also reported the status of 55 ablation zones immediately after ablation and 1 day after ablation and showed that the immediate low echogenicity of the treatment area was converted into total hyper-echogenicity on the 1st postoperative day [19]. Appelbaum et al. speculated that red blood cells progressively infiltrated into deep regions of the ablated zone such that the hyper-echoic rim on US at 90–120 min transitioned to complete hyper-echogenicity within 24 h [5]. Concordance exists between our findings and those of previous studies describing the characteristics of images obtained after ablation.

In CEUS, most of the ablated zones showed hypo-enhancement immediately after IRE. Chung et al. found the following zones with different enhancement patterns on CT perfusion images in normal porcine liver: an inner non-enhanced zone; a middle well-defined progressive internal enhancement zone, and an outer ill-defined arterial enhancement zone. On histopathology, the inner and middle zones accounted for the extent of cell death [20]. The histological examination suggested that the apoptotic process was involved, with complete cell death caused by IRE according to pathophysiology.

The CEUS images of the IRE ablation zones in normal liver tissue differed from the images of liver tumours treated with IRE. Our study also found that 12 ablated zones showed hypo-enhancement immediately after IRE but became non-enhanced one day after IRE for various reasons. Lee et al. attributed the focal hyper-attenuation to the release of contrast medium into an ablation defect caused by an IRE-induced microvasculature leakage within the defect zone [21]. Because the zones became non-enhanced on the follow-up CEUS images, the hyper-enhancement was probably caused by extraluminal contrast material. Another opinion concerns differences in contrast agent concentrations. Guo et al. suggested that nanometre-scale pores in the tissue cell membrane caused contrast agent to accumulate in the IRE zone and allowed the contrast agent to be internalized into the intracellular environment rather than remaining extracellularly [22], which complicates evaluation of whether the viable tissue is a residual tumour after ablation. Therefore, follow-up CEUS is necessary to assess the viable portion. However, unlike normal regeneration activity, the residual tumour can continue growing and results in a newly enhanced region.

Most of the non-enhancement pattern on CEUS 1 month after ablation is a sign of effective IRE treatment, but false-negative results may occur. Such non-enhancement patterns are different to those of the thermal ablation zone after complete ablation. Previous histologic examinations of the ablation zones showed cell death caused by apoptosis [19]. Cell death is observed with full preservation of the peri-ablative zone structures, such as blood vessels and bile ducts.

In the present study, only one patient continuously showed an enhanced ablation zone immediately and on follow-up CEUS images. The ablation zone became hyper-enhanced during the early arterial phase, slightly washed out in the late arterial phase and appeared hypo-enhanced in the late phase. To elucidate the reason for this pattern, the patient underwent liver biopsy one month after IRE. The physiology results showed small patchy necrosis, inflammatory fibrosis, tissue hyperplasia and foam cell aggregation. The result ruled out the possibility of recurrence. A reasonable explanation for this finding is hyperplasia of the inflammatory tissue. This result can cause confusion in clinical practice. Therefore, a sequential follow-up is essential. Further investigation is needed to study the histological and cytological mechanisms underlying this process.

In our study, only 2 ablation zones still appeared as hypo-echoic areas, and both zones were in the same patient. After 1 month of follow-up, recurrence occurred near one of the zones treated with IRE ablation. In a previous multi-institutional review from 2009 through 2012, 31% of the patients had recurrence during a median follow-up of 18 months. Among the study population, 31% of the patients had recurrence, and 10.7% had local recurrences at the ablated site [4]. Previous studies have attributed such recurrences to electric field sinks resulting from the heterogeneous structure and conductivity of the liver [23]. Later work from the authors indicated that the IRE-treated extracellular matrix (ECM) provides an environment for activation and differentiation of progenitor cells [24], but the mechanism is not completely understood; in contrast, some studies have suggested that abnormal ECM affects cancer progression by directly promoting cellular transformation and metastasis as well as tumour-associated angiogenesis and inflammation, leading to the generation of a tumorigenic microenvironment [25]. The role of the IRE-spared tumour matrix in follow-up recurrences requires further research. Another theory is related to the size of the treated liver tumours. Niessen et al. found that large tumour volumes (> 5 cm^3^) portended early local recurrence [26].

In our study, the intra-hepatic blood vessels and bile duct remained almost completely intact after IRE. The hepatohilar bile duct of only one patient showed an unclear contrast agent pattern immediately after IRE. After 15 min, the phenomenon disappeared. However, a consensus on the mechanism of IRE has not yet been reached. This effect may be due to the high proportion of collagenous connective tissue. A further hypothesis is that gap junctions, which are present in large numbers in the muscularis propria of blood vessels and the bile duct walls, may act as a conductive structure for the electrical currents; thus, the current can pass from cell to cell without causing destruction of the cell membrane [14]. Whether the effects of IRE are caused by a thermal or non-thermal mechanism remains unclear.

This study has several limitations. First, our study had a small sample size over a span of more than 2 years. Second, 9 lesions of the tumour were not diagnosed histologically before IRE. The last limitation was that almost all of the patients included in our study group previously underwent right or left liver resection and thermal ablation or catheter chemical ablation. The origin of the ablation zones was heterogeneous. However, this circumstance mirrors the status of our clinical treatment strategies. IRE as a novel technique was not the first choice in our hospital. Only patients who experienced tumour recurrence after liver resection and thermal ablation were considered for IRE. Because of this situation, assessing the images of the ablated zones during subsequent follow-up imaging examinations is important. We conclude that CEUS may be a useful tool for assessing the characteristics of post-IRE ablation changes and is an effective method to evaluate the therapeutic efficacy 1 month after ablation. Further studies are needed to evaluate more patients to precisely depict the appearance of hepatic zones on CEUS.

## Data Availability

The datasets used and/or analysed during the current study are available from the corresponding author upon reasonable request.

## Abbreviations

IRE: Irreversible electroporation
CEUS: Contrast-enhanced ultrasound
US: Ultrasound
MRI: Magnetic resonance imaging
RFA: Radiofrequency ablation
MWA: Microwave ablation
ECM: Extracellular matrix
